# Chaotic Micromixer Based on 3D Horseshoe Transformation

**DOI:** 10.3390/mi10060398

**Published:** 2019-06-14

**Authors:** He Zhang, Xin Li, Rongyan Chuai, Yingjie Zhang

**Affiliations:** School of Information Science and Engineering, Shenyang University of Technology, Shenyang 110870, China; lixin@sut.edu.cn (X.L.); chuairongyan@sut.edu.cn (R.C.); zyj13322427535@126.com (Y.Z.)

**Keywords:** microfluidic, chaotic mixing, 3D horseshoe transformation, mass transfer

## Abstract

To improve the efficiency of mixing under laminar flow with a low Reynolds number (Re), a novel three-dimensional Horseshoe Transformation (3D HT) was proposed as the basis for the design of a micromixer. Compared with the classical HT, the Lyapunov exponent of the 3D HT, which was calculated based on a symbolic dynamic system, proved the chaotic enhancement. Based on the 3D HT, a micromixer with a mixing length of 12 mm containing six mixing units was obtained by sequentially applying “squeeze”, “stretch”, “twice fold”, “inverse transformation”, and “intersection” operations. Numerical simulation and Peclet Number (Pe) calculations indicated that when the squeeze amplitude 0 < *α* < 1/2, 0 < *β* < 1/2, the stretch amplitude *γ* > 4, and Re ≥ 1, the mass transfer in the mixer was dominated by convective diffusion induced by chaotic flow. When Re = 10, at the outlet of the mixing chamber, the simulated mixing index was 96.4%, which was far less than the value at Re = 0.1 (*σ* = 0.041). Microscope images of the mixing chamber and the curve trend of pH buffer solutions obtained from a mixing experiment were both consistent with the results of the simulation. When Re = 10, the average mixing index of the pH buffer solutions was 91.75%, which proved the excellent mixing efficiency of the mixer based on the 3D HT.

## 1. Introduction

In addition to drive pressure, passive micromixers do not require external excitation and are therefore more suitable for integration in micro total analysis systems (μTASs) [1,2]. The characteristic scale of a μTAS is 0.1 μm to 1 mm, and the fluid generally moves under laminar flow with a low Reynolds number (Re) [3]. It is necessary to operate the fluid with the help of special microstructures to improve mixing. The simplest structures are the T-shape [4] and the Y-shape [5], which mainly utilize the diffusion motion between molecules to achieve mixing. However, the mixing efficiency cannot easily meet the requirements of a μTAS. The split-and-recombine (SAR) micromixer, which was designed to increase the area of fluid contact surfaces, is another typical passive mixer [6,7]. Although the expected mixing efficiency can be obtained after repeated SAR operations, the excessive mixing distance means that this mixer is not suitable for μTAS. Obstacles in the micro-channel can improve the mixing efficiency [8]; however, the drive pressure drops sharply, which affects other modules in the μTAS.

Chaotic fluid not only maintains the basic characteristics of laminar flow, such as low velocity and small pressure drop, but its diffusion characteristics are closer to turbulent flow, which can significantly improve mixing efficiency [9,10]. Special geometries, such as serpentine [11], twisted [12], Tesla [13], etc. [14,15], can also generate chaotic fluid. However, the design process relies extensively on experience, which results in uncertain mixing performance. The staggered herringbone [16], which uses the interlaced structure at the bottom of the micro-channel to induce chaotic flow under low Re, is a representative chaotic mixer design principle. Song et al. optimized the staggered herringbone mixer by replacing the three-dimensional (3D) bottom structure with a two-dimensional (2D) structure [17]. This improved the mixing efficiency while reducing the preparation difficulty. However, the optimized mixer still requires 15 cycles (mixing distance = 27 mm) to obtain satisfactory results. Another mixer design instruction, which subjects the fluid to a sequence of “squeeze”, “stretch”, “cut”, and “stack” (SSCS), was based on Baker’s Transformation (BT) [18,19]. Yasui et al. fabricated the first BT micro-mixer, which repeated 10 SSCS operations in a length of 10.4 mm [20]. Peter et al. performed multiple “cutting and stacking” operations simultaneously by using a semi-parallel structure, which could promote the mixing efficiency to grow exponentially [21]. Although the performance of the BT mixer is excellent, its structures are very complicated due to the “cut” and “stack” operations, which significantly increases the integration difficulty.

The Horseshoe Transformation (HT) [22,23], which uses a “fold” operation to replace the “cut” and “stack” operations of Baker’s Transformation, can also guide the design of a micromixer [24,25,26]. However, the HT is isomorphic with Bernoulli only under specific conditions, such as zero volume [27]. Therefore, compared with BT mixers, the formation conditions of high-intensity chaotic flow, which could determine mixing efficiency, become more stringent. In this paper, a 3D HT is presented by increasing the “secondary fold” operation. The improvement in chaotic intensity provided by the 3D HT could be proven by calculating with the symbolic dynamic system. Then, a micromixer that accorded with the 3D HT was designed and assembled. The enhancement of mixer efficiency was verified by numerical simulation, optical microscopy observation, and the adjustment of buffer solution pH, respectively. The micromixer design process based on the mathematical model could ensure chip performance.

## 2. Method

**Figure 1:**
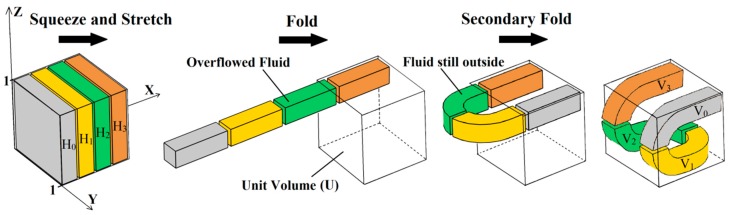
The three-dimensional (3D) Horseshoe Transformation (HT) acting on the unit volume.

The 3D HT is shown in Figure 1. When the unit volume (*U*) in the *R*^3^ is defined as *U* = [0, 1] × [0, 1] × [0, 1], the original fluid in *U* can be distinguished by four blocks: *H*_0_ (grey), *H*_1_ (golden), *H*_2_ (green), and *H*_3_ (brown).

The mathematical expression of the original fluid in *U* is obtained by using the following four elements of the symbolic dynamic system:

(1) $$\begin{array}{l} H_{0}=\{(x,y,z)\in R^{3}|0\leq x<\frac{1}{4},0\leq y\leq 1,0\leq z\leq 1\}\\ H_{1}=\{(x,y,z)\in R^{3}|\frac{1}{4}<x<\frac{1}{2},0\leq y\leq 1,0\leq z\leq 1\}\\ H_{2}=\{(x,y,z)\in R^{3}|\frac{1}{2}<x<\frac{3}{4},0\leq y\leq 1,0\leq z\leq 1\}\\ H_{3}=\{(x,y,z)\in R^{3}|\frac{3}{4}<x\leq 1,0\leq y\leq 1,0\leq z\leq 1\} \end{array}$$

The fluid is squeezed along the Y-axis and the Z-axis, the compression ratio in the Y direction is *α* (0 < *α* < 1/2), and the compression ratio in the Z direction is *β* (0 < *β* < 1/2). Due to its incompressibility, the fluid is stretched along the X-axis. The stretch ratio in the X direction is *γ* (*γ* > 4). After squeezing and stretching, the original fluid is moved into four slender blocks, and overflow U occurs in the X direction. The overflowed fluid is then folded back into *U* in the X-Y plane. However, some fluid still remains outside *U* after the folding. Then, the fluid remaining outside is folded back into *U* in the X-Z plane. After folding twice, the amount of fluid that remains outside *U* is reduced drastically. The fluid blocks (*V*_0_, *V*_1_, *V*_2_, and *V*_3_) that are subjected to squeezing, stretching, and double folding processes can be described by a mapping *f:* *U*→ *R*^3^ as in Equation (2). Here, *N_i_*, *M_i_*, and *L_i_* (*i* = 0, 1, 2) are constant, and the negative sign indicates that the direction of the fluid flow is changed.

(2) f(H0)≡V0:[xyz]↦[−γ000−α000β][xyz]f(H1)≡V1:[xyz]↦[−γ000−α000−β][xyz]+[N0N1N2](0<α<12;0<β<12;γ>4)f(H2)≡V2:[xyz]↦[−γ000α000−β][xyz]+[M0M1M2]f(H3)≡V3:[xyz]↦[γ000α000β][xyz]+[L0L1L2]

After mapping *f*, the fluid that is still in *U* can constitute a set as in Equation (3). The set *V_j_* within *U* consists of four disjointed “fluids”, which are parallel to the X-Y plane.

(3) $$V_{i}=U\cap f(H_{i})\;(i=0,1,2,3)$$

**Figure 2:**
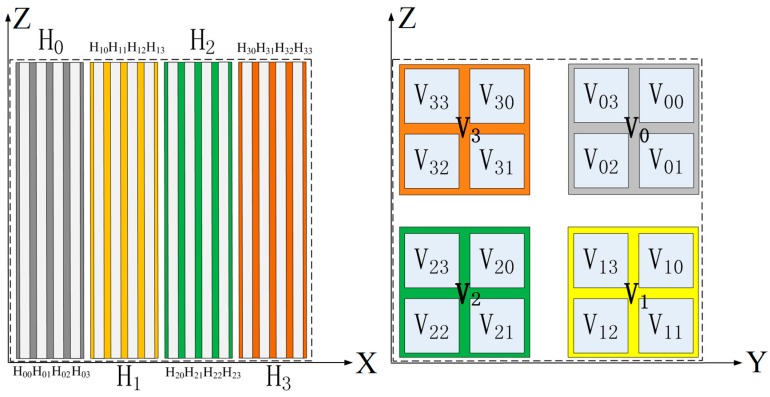
Schematic of the 3D HT under mapping *f*^2^.

Repeat the squeezing, stretching, and twice folding operations. The compression ratio in the Y direction is denoted as *α*^2^, the compression ratio in the Z direction is denoted as *β*^2^, and the stretch ratio in the X direction is denoted as *γ*^2^. A schematic of the 3D HT under mapping *f*^2^ is shown in Figure 2. In order to show the mapping of *f*^2^ clearly, the projections on the X-Z plane and the Y-Z plane are given in the figure, respectively.

After mapping *f*^2^, the fluid that is still in *U* becomes 4^2^ more slender blocks and can be described by Equation (4):

(4) $$f(V_{ij})=U\cap f(U\cap f(H_{i}))=U\cap f(U)\cap f^{2}{\left(H_{i}\right)}_{}^{}\;(i,j=0,1,2,3)$$

When *n*th iteration mapping is reached, the fluid that is still in *U* can be described by Equation (5):

(5) $$\Lambda_{n\to \infty }=U\cap f(U)\cap f^{2}(U)\cdots f^{n-1}(U)$$

The invariant set of invertible mapping can be constructed by using a similar method, as described in Equation (6):

(6) $$\Lambda_{n\to -\infty }=U\cap f^{-1}(U)\cap f^{-2}(U)\cdots f^{-(n-1)}(U)$$

Thus, the invariant set *Λ* of mapping *f:* *U*→ *R*^3^, which has a self-similar structure, can be given by Equation (7):

(7) $$\Lambda =\Lambda_{-\infty }\cap \Lambda_{+\infty }=\overset{\infty }{\underset{n=-\infty }{\cap }}f^{n}(U)$$

Obviously, the 3D HT can be attributed to a dynamic system (*f*, *Λ*). According to the topological conjugacy of dynamical systems, if (*f*, *Λ*) is chaotic, the 3D HT is also chaotic. In order to prove the chaoticness of dynamic system (*f*, *Λ*), the Lyapunov exponent *L*(*P*; *f*) is introduced by Equation (8) [28]. Here, *D* is the Jacobian of the mapping, while *P*(*x*, *y*, *z*) are arbitrary points in the unit volume *U*:

(8) $$L(P;f)=\underset{n\to \infty }{\lim}{\left(Df^{n}{\left(x,y,z\right)}^{T}Df^{n}(x,y,z)\right)}^{\frac{1}{2n}}$$

As a ubiquitous diagnostic in chaotic dynamics, the Lyapunov exponent can not only describe orbit changes but can also characterize the chaotic intensity. When *L*(*P*; *f*) < 0, the separation between particles decreases exponentially, and the dynamic system (*f*, *Λ*) is non-chaotic and insensitive to the initial value; when *L*(*P*; *f*) > 0, the particle orbits are rapidly separated at an exponential rate, and the system is sensitive to the initial value and can generate chaos; when *L*(*P*; *f*) = 0, the particle orbits neither shrink nor separate, and the system is in a critical state [29]. Combining Equation (2), the three Lyapunov exponents of dynamic system (*f*, *Λ*) can be described by Equation (9). As ln|γ|>0, the dynamic system (*f*, *Λ*) is chaotic, and the 3D HT is also chaotic:

(9) ln|α|<0(0<α<12)ln|β|<0(0<β<12)ln|γ|>0(4<γ)

According to references [22,23,28], the two Lyapunov exponents of the classic Horseshoe Transformation can be calculated by Equation (10). Obviously, the chaotic intensity of the 3D HT is greater than that of the classic HT, since ln|γ|>ln|μ|(γ>4;μ>2):

(10) ln|λ|<0 (0<λ<12)ln|μ|>0 (2<μ)

## 3. Mixer Design and Manufacture

**Figure 3:**
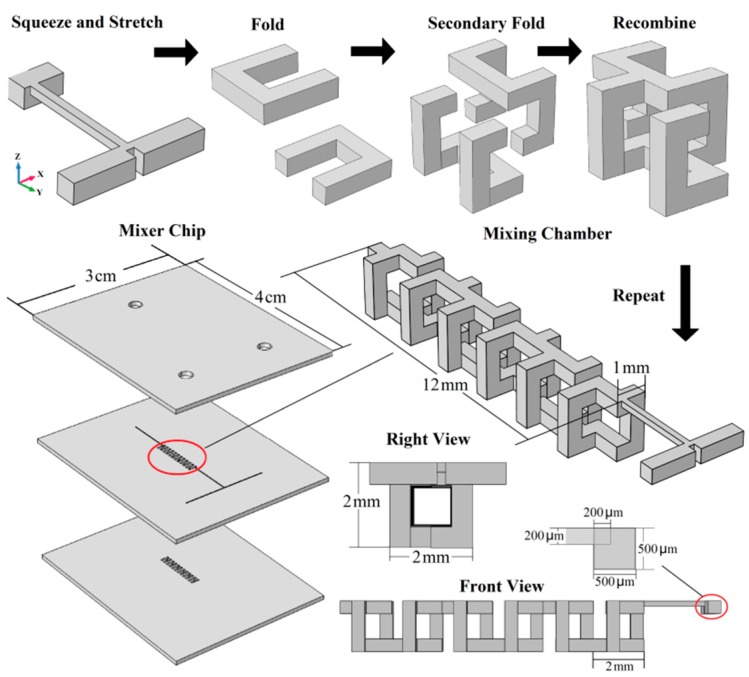
Design process and mixer structure based on the 3D HT.

According to the 3D HT mapping, the mixer design processes and the chip structure are shown in Figure 3. The design processes can be summarized as follows. When the two fluids met in the T-type micro-channel, the cross-sections changed from 500 × 500 μm to 200 × 200 μm, which satisfied the squeezing condition in the Y direction and the Z direction (0 < *α* < 1/2; 0 < *β* < 1/2). When confluence fluid entered the mixing chamber, the length of the micro-channel changed from 200 to 1000 μm in the X direction, which satisfied the stretching condition of the 3D HT (*γ* > 4). After squeezing and stretching, the fluid was folded in different directions according to the 3D HT and was then recombined to constitute one mixing unit. Finally, a mixing chamber with a length of 12 mm with six mixing units was obtained. The chip with the mixing chamber as its core consisted of a cover layer with an inlet and an outlet, an intermediate layer with a T-shaped inlet channel and a portion of the mixing chamber, and a back sheet layer with the remaining portion of the mixing chamber.

**Figure 4:**
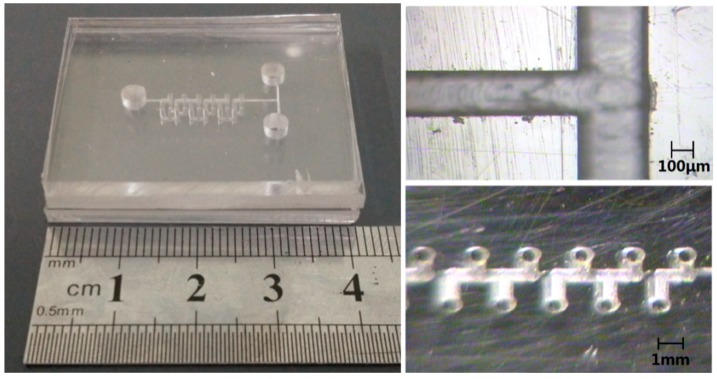
Photographs of the mixer chip.

Polymethyl methacrylate (PMMA; Stone into Gold Trading Co., Ltd., Dongguan, China) was chosen as the mixer chip material. The micro structures were manufactured using a micro-precision engraving machine (VIP3530; Thai Power Electronic Equipment (Beijing) Co., Ltd., Beijing, China). Ultrasonic cleaning equipment (KQ-5200DB; Kun Shan Ultrasonic Instruments Co., Ltd., Kunshan, China) was used for substrate cleaning. The miscible organic solvents soak bonding method [30] was used to bond the multilayer mixing device. The bonding processes are summarized below. First, a miscible solution of chloroform and ethanol (volume ratio 1:10) was formulated for use as the bonding solvent. Second, in the miscible solution, the substrates were aligned and fixed by using a quartz glass fixture and nylon screws. Finally, the bonding interface was immersed into a miscible solution and then put into a vacuum oven (DZF-6020; Shanghai Jing Hong Laboratory Instrument Co., Ltd., Shanghai, China) and heated for 10 min at 40 °C to complete the bonding. To avoid extra damage to the microstructure, only one layer was bonded at a time. Photographs of the assembled chip are shown in Figure 4.

## 4. Results and Discussion

### 4.1. Performance Simulation

Before simulation, Re can be calculated by Equation (11). When the fluid density (*ρ*) is 1 × 10^3^ kg/m^3^, the mixing chamber structural characteristic scale (*L*) is 5 × 10^−4^ m, the flow velocity (*u*) is 2 × 10^−2^ m/s, and the dynamic viscosity coefficient (*η*) is 1 × 10^−3^ Pa·s, Re=10≪2300, which proves that the fluid movement in the micromixer is a typical laminar flow:

(11) $$Re=\frac{\rho uL}{\eta }$$

In a laminar flow condition, the fluid flow of the model is described by the Navier–Stokes equations as follows:

(12) $$\begin{array}{c} \rho u\cdot \nabla u=-\nabla p+\nabla \mu (\nabla u+{\left(\nabla u\right)}^{T})\\ \nabla \cdot u=0 \end{array}$$

where *ρ* is the density (kg/m^3^), *u* is the velocity (m/s), *μ* is the viscosity (N·s/m^2^), and *p* is the pressure (Pa). The modeled fluid is water with a viscosity of 1 × 10^−^^3^ N·s/m^2^ and a density of 1000 kg/m^3^. The mass transport of the model is described by the convection–diffusion equation as follows:

(13) $$D\nabla^{2}c-u\nabla c+R=0$$

where *D* is the diffusion coefficient (m^2^/s), *c* is the concentration of the components (mol/m^3^), and *R* is the reaction rate between components. When no reactions occur between the fluids to be mixed, *R* = 0, and the mass transport between fluids is determined using both convective diffusion (u∇c) and molecular diffusion ($D\nabla^{2}c$).

Based on the above equations, a series of simulations were executed by using COMSOL Multiphysics. Additionally, the simulation model included the following specific assumptions:

1. The fluid in the model was incompressible and Newtonian;
2. There was no chemical reaction between the fluids;
3. There were no slip boundary conditions;
4. We did not consider fluid infiltration, bubbles within the fluid, or fluid polarity.

Orderly and clearly unstructured tetrahedral units were chosen as mesh elements. The independence of the number of mesh elements was calculated based on the results of visual tests, and the concentration distribution of the mixing chamber surface was predicted by the simulation results. The microscopic images and the simulation results for surface concentration distribution for different numbers of mesh elements were converted to grayscale using the open source image processing software ImageJ (National Institutes of Health, Bethesda, MD, USA). The number of gray pixels in each image was counted to compare the simulation accuracy and determine the mesh independence. A comparison of the accuracy for different numbers of mesh elements is shown in Table 1. In the table, the number of mesh elements is divided into five levels, namely coarser, coarse, normal, fine, and finer. When the number of mesh elements increased from coarser (5.8 × 10^3^) to fine (87.2 × 10^3^), the relative error of the model dropped from 34.45% to 5.52%. When the number of mesh elements became finer (254.26 × 10^3^), the relative error of the model dropped to 5.43%, and the calculation time was more than five hours using a Lenovo 510 pro computer with an Intel i5-9400F processor and 8 GB of DDR4 RAM. Therefore, in order to ensure simulation efficiency while limiting the relative error, the mixing chamber structure was divided by fine-level (87.2 × 10^3^) mesh elements (calculation time = 97 min).

The underlying finite element discretization method used in this model was the Galerkin method. When the mass transport equation was discretized using the Galerkin method, the resulting numerical problem became unstable if the Peclet Number (Pe) was larger than one. Therefore, some other techniques were required to further ensure numerical stability without mesh refinement in the model. Consistent stabilization methods that did not perturb the original mass transport equation were selected. These methods included streamline diffusion and crosswind diffusion. Streamline diffusion introduces artificial diffusion in the streamline direction. It is often sufficient to obtain a smooth numerical solution if the exact solution of mass transport does not contain any discontinuities. However, undershoots and overshoots can occur in the numerical solutions when sharp gradients are present. The crosswind diffusion that introduces orthogonal diffusion to the streamline direction can address the above spurious oscillations. Therefore, streamline diffusion and crosswind diffusion should be applied at the same time to further ensure numerical stability.

**Figure 5:**
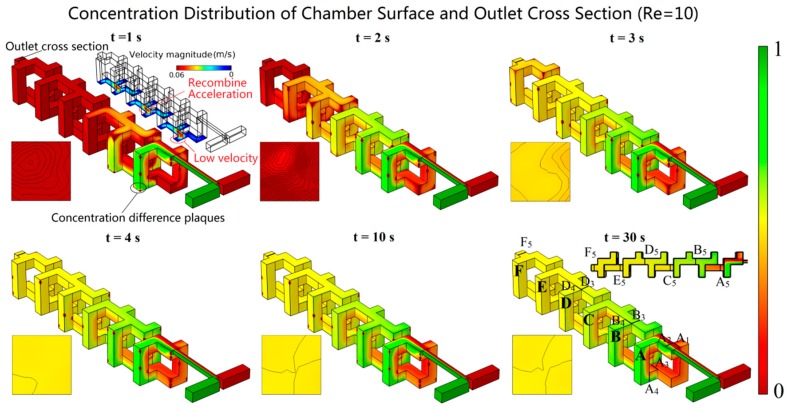
The concentration distribution of the mixing chamber surface and the outlet cross-section at different times.

Figure 5 shows the concentration distributions of the mixing chamber surface and the outlet cross-section obtained using the above model. The fluids for mixing are shown in green (1 mol/L) and red (0 mol/L), while the color gradient between them indicates the degree of mixing. The concentration contours had increments of 0.02 mol/L. From Figure 4, it can be seen that when *t* = 1 s, the fluids to be mixed only passed through two mixing units, and the color of the outlet cross-section was red (i.e., the concentration was 0 mol/L). When *t* = 2 s, the fluids passed through four mixing units, and the color of the outlet cross-section was close to red, which indicated that the concentration of the outlet cross-section was close to 0 mol/L. When *t* = 3 s, the fluids almost passed through the entire mixing chamber, and the color of the outlet cross-section was close to yellow (0.5 mol/L), which was the center of the color gradient. Moreover, the interface between the two fluids was difficult to distinguish near the outlet of the mixing chamber. When *t* = 4 s, the fluids passed through the entire mixing chamber, the color of the outlet cross-section was a uniform yellow, and the interface between the two fluids was indistinguishable. When *t* > 4 s, the mixing chamber surface and the outlet cross-section concentration distribution were not significantly changed, and the mixer worked in a stable state. However, there were many red plaques at the corners and the edges of the mixer structure. A large difference in concentration was observed between these plaques and the surrounding fluid. From the velocity magnitude distribution shown in Figure 5, it can be seen that when Re = 10, the fluid velocity at the corners and the edges was much lower than that at the center of the structure, even close to 0 m/s. These almost stationary fluids could only be mixed by molecular diffusion. Therefore, it took more time to eliminate these plaques. When *t* = 30 s, the plaques completely disappeared due to sufficient molecular diffusion.

**Figure 6:**
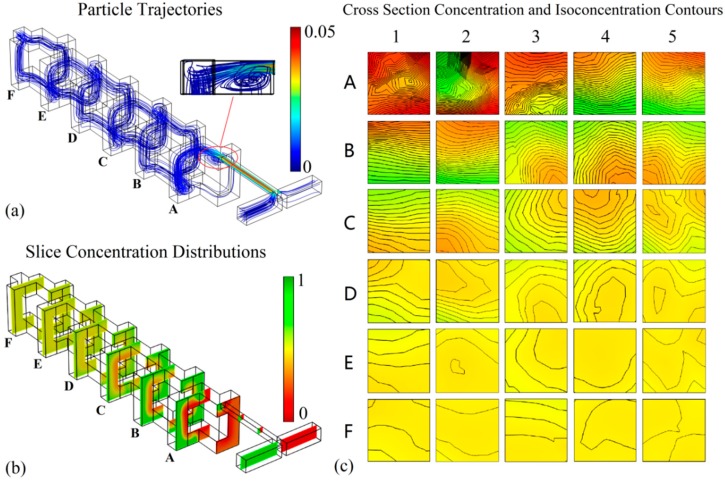
The internal mixing situation of the 3D HT mixer. (**a**) Particle trajectories of 3D HT; (**b**) Slice concentration distribution inside 3D HT; (**c**) Concentration and isoconcentration contours of 3D HT cross-section.

In order to investigate the mixing inside the device at *t* = 30 s when Re = 10, the particle trajectories, the slice concentration distributions, and the cross-section isoconcentration contours are shown in Figure 6. The cross-sections, which included folding before (A_1_, A_2_, B_1_, B_2_, C_1_, C_2_, D_1_, D_2_, E_1_, E_2_ and F_1_, and F_2_), folding after (A_3_, A_4_, B_3_, B_4_, C_3_, C_4_, D_3_, D_3_, E_3_, E_4_ and F_3_, and F_4_), and recombination (A_5_, B_5_, C_5_, D_5_, E_5_ and F_5_), are shown in Figure 5. As can be seen from Figure 6a, for relatively high Re (Re = 10), there was a significant vortex effect inside the mixer. The vortexes were generated because the sudden expansion of the structure led to the differentiation of the flow rate when the fluids flowed out of the outlet of the T-shaped channel [31]. The fluids were accelerated once more due to the recombination operation at the outlet of mixing unit A. When the accelerated fluids entered mixing unit B, the flow rate differentiated again. The repetition of the mixing unit structure led to the periodic generation of vortexes. As shown in Figure 6b, in mixing unit A, the interface between the two fluids was clear. In mixing units B and C, the interface of the two fluids was blurred. In mixing unit D, the interface of the two fluids was indistinguishable. In mixing units E and F, the fluids became a uniform yellow color. The isoconcentration contours before and after the folding operation are shown in Figure 6c. In A_1_, the cross-section that was closest to the inlet of the mixing chamber, the red color was dominant. In A_2_, another no-fold cross-section, red and green colors were present, and the interface between them was sharpened. In A_3_, which was obtained by the double folding of cross-section A_1_, red still dominated; however, the proportion of yellow was significantly increased. In A_4_, which was obtained by the double folding of cross-section A_2_, the gradient between the two colors was macroscopically decreased. In A_5_, which was the cross-section after the first recombination, the color gradient was further reduced; however, the interface was still clear. Additionally, the isoconcentrations on A_1_ and A_2_ were very dense, and it seemed that some of them overlapped. The isoconcentrations on A_3_ and A_4_ were still dense, but they were not as crowded. The isoconcentrations on A_5_ were further decreased. There was a certain distance between each isoconcentration. In each cross-section of mixing units B, C, and D, the color gradient of the concentration cloud and the number of isoconcentrations were reduced step by step, which illustrated the continuous improvement of fluid uniformity. In each cross-section of mixing units E and F, the change of the color gradient was not obvious, and the interface between the two colors was difficult to distinguish. The number of isoconcentrations in all the cross-sections of mixing units E and F was fewer than five, which illustrated that the concentration difference was not only very small but also steady.

However, the particle trajectories, the slice concentration distribution, and the number of isoconcentrations could only describe the improvement of mixer performance roughly, and it was impossible to prove whether chaotic flow occurred inside the mixer. In order to accurately quantify the mixing effectiveness, the mixing concentration variance (*σ*) in Equation (14) was used:

(14) $$\sigma =\sqrt{\frac{1}{N}\sum_{i=1}^{N}{\left(C_{i}-\bar{C}\right)}^{2}}$$

where *C_i_* is the concentration of the statistical area, *N* = 10 is the number of samples in the statistical area, and $\bar{C}$ = 0.5 mol/L is the average concentration.

**Figure 7:**
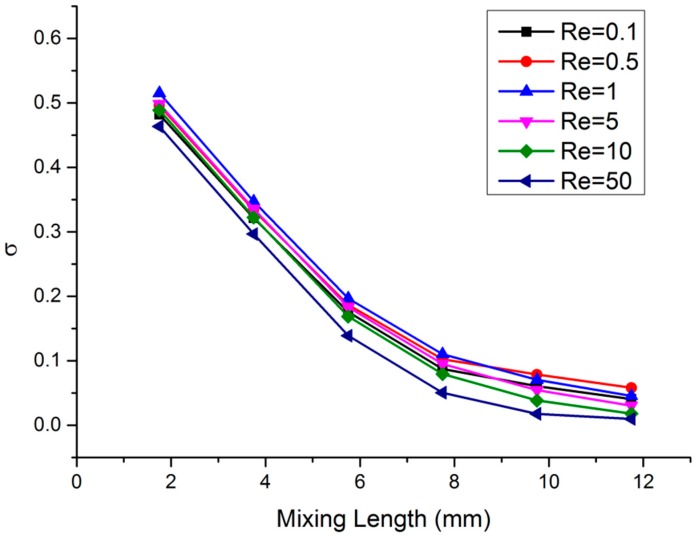
Concentration variance (*σ*) for different Reynolds number (Re) in the 3D HT mixer.

Figure 7 shows the curves of concentration variance (*σ*) against Re when sampling points were at the recombination cross-sections (A_5_, B_5_, C_5_, D_5_, E_5_, and F_5_). From the figure, it can be seen that when Re ≤ 0.5, *σ* was proportional to Re, the mixing efficiency was inversely proportional to Re. When Re = 0.1, the concentration variance was *σ =* 0.041 at the outlet of the mixing chamber (cross-section F_5_). Since the mixing chamber’s characteristic structural scale was *L* = 5 × 10^−4^ m, the flow velocity *u* = 2 × 10^−^^4^ m/s could be calculated by Equation (11). Then, when the diffusion coefficient (*D*) was 1 × 10^−9^ m^2^/s, the mixing chamber’s characteristic structural scale (*L*) was consistent, and the Peclet Number Pe = 1 could be calculated by Equation (15):

(15) $$Pe=\frac{uL}{D}$$

According to the result of the Pe calculation, the mass transfer in the mixing chamber was mainly determined by molecular diffusion. The effectiveness of molecular diffusion was related to the contact area and the contact time between the fluids to be mixed. The contact area was constant, since the mixing unit structure was identical. When the flow velocity increased with Re, the contact time and the mixing efficiency decreased. When Re = 1, after the fourth recombination (cross-section D_5_), the concentration variance (*σ* = 0.110) was still higher than the value at Re = 0.5 (*σ* = 0.102). However, after the fifth recombination (cross-section E_5_), the concentration variance (*σ* = 0.071) was less than the value at Re = 0.5 (*σ* = 0.079), and the trend of the curves turned over. When Re = 1, the flow velocity *u* = 2 × 10^−3^ m/s, and Pe = 10 could be calculated by Equation (15), which proved that the mass transfer in the mixing chamber was determined by both molecular diffusion and convection. When Re = 5, at the outlet of the mixing chamber (cross-section F_5_), the concentration variance (*σ* = 0.030) was less than the value at Re = 0.1 (*σ* = 0.041). When Re = 10, at the outlet of mixing unit C, the concentration variance *σ* = 0.169 was less than the value at the same position when Re = 0.1 (*σ* = 0.175). At the outlet of the mixing chamber (cross-section F_5_), *σ* = 0.0181 (the mixing index *α* = 96.4%), which was far less than the value at Re = 0.1 (*σ* = 0.041). Additionally, a significantly increased Re = 50 was chosen to investigate the influence of inertia on mixing. When Re = 50, at the outlet of mixing unit A (cross-section A_5_), the concentration variance was *σ* = 0.464, which was the lowest value at this position. Meanwhile, at the outlet of the mixing chamber (cross-section F_5_), *σ* = 0.00965 (the mixing index *α* = 98.07%), which was the best simulation mixing efficiency obtained in this paper.

The above data confirmed that the convection contributed increasingly to mass transfer when Pe increased with flow velocity and Re. However, the mixer could not easily produce convective diffusion without external driving if the fluid’s only motion was laminar with a low Re. Therefore, it could be inferred that chaotic flow, which is the cause of convective diffusion, was generated inside the mixer.

### 4.2. Performance Test

Before the performance test, Re was converted to volumetric flow *Q* (m^3^/s) according to Equation (16):

(16) Q=ReLη0ρ

where *L* is the side length of the equivalent square channel, *η_0_* is the viscosity of the dyed water (8 × 10^−4^ Pa·s at 25 °C), and *ρ* is the density of water (998 kg/m^3^). The correspondence between Re and the volumetric flow rate was as follows: when Re = 0.1, *Q* = 0.024 mL/min; when Re = 0.5, *Q* = 0.12 mL/min; when Re = 1, *Q* = 0.24 mL/min; when Re = 5, *Q* = 1.2 mL/min; and when Re = 10, *Q* = 2.4 mL/min.

**Figure 8:**
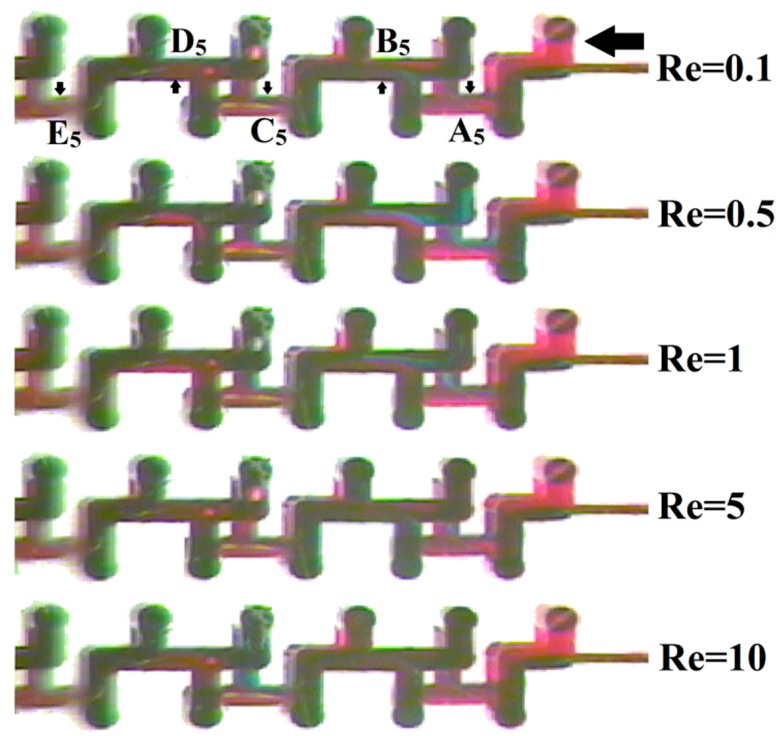
Microscopic images of the mixing chamber under different Re.

Then, a visualization test was conducted to intuitively observe the 3D HT mixer process. Rhodamine B (red) and Methyl Green (bluish-green) dyes were utilized as indicators. A dual-channel micro-injection pump (SN-50F6; Sino Medical-Device Technology Co., Ltd., Shenzhen, China) was used to provide impetus and controlled volumetric flow, and a microscope (C3203A; Shanghai Precision Instrument Co., Ltd., Shanghai, China) was used to observe the mixing progress under different Re. Microscopic top-view images of the 3D HT mixer are shown in Figure 8. The arrow indicates the direction of fluid flow, and the positions of the letters (A_5_, B_5_, C_5_, D_5_, and E_5_) are consistent with recombination cross-sections in the concentration distribution of Figure 5. Due to the limitation of the microscope’s field of view, the recombination cross-section F_5_ was not visible in the images; however, the five mixing units were sufficient to verify the mixer performance. From the images, it can be seen that when Re = 0.1, at cross-section B_5_, the fluid interface could be easily identified after the double-folding and the recombination operations, while the interface became indistinct after the fourth recombination (cross-section D_5_). When Re = 0.5, at cross-section B_5_, the interface between the two fluids became clearer, and the fluid interface could still be easily identified in cross-section D_5_; all of the above changes proved the decline in mixing efficiency. When Re = 1, at cross-sections B_5_ and D_5_, the fluid interface became harder to identify but not significantly so. When Re = 5, at cross-section B_5_, the fluid interface was not visible, while the fluid interface was invisible at cross-section D_5_. When Re = 10, at cross-section B_5_, the fluid interface was invisible, which indicated further improvement of the mixing efficiency. The results of the visualization test indicated that the variation trend of mixing efficiency under different Re was consistent with the simulation.

Next, standard pH buffer solutions (INESA Scientific Instruments, Ltd., Shanghai, China) containing potassium hydrogen phthalate (0.05 mol/L, pH = 4.01), mixed phosphate (0.025 mol/L, pH = 6.86), and borax (0.01 mol/L, pH = 9.18) were prepared at room temperature (25 °C) to more accurately test the mixing performance. To provide a reference, any two equal-volume solutions (10 mL) were homogeneously mixed using a magnetic stirrer. The obtained buffer solutions were calibrated using a pH meter (JB-1A; INESA Scientific Instruments, Ltd., Shanghai, China). Each measurement was repeated three times, and the values were averaged. The results of the calibration are shown in Table 2.

**Figure 9:**
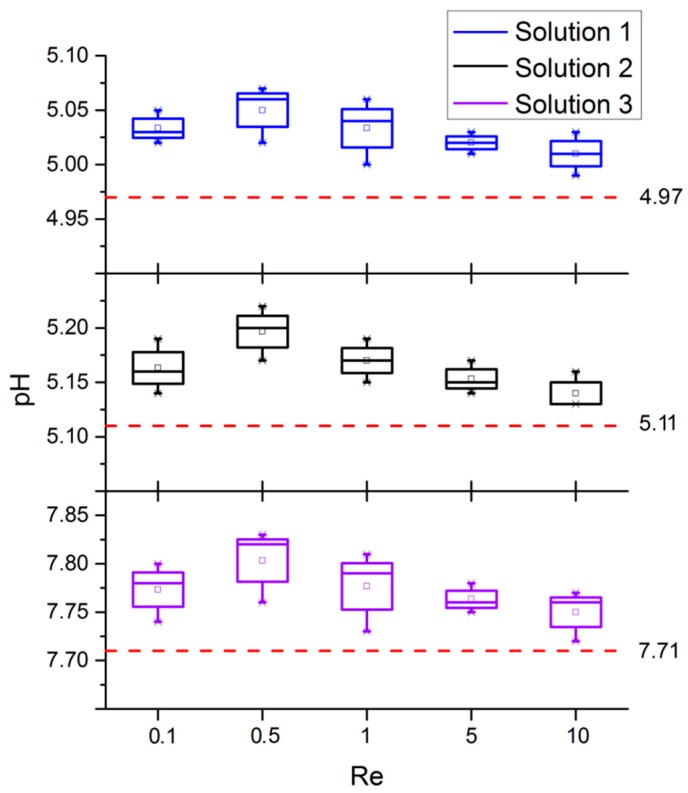
Test results for the pH of buffer solutions for various Re.

After the calibration of reference solutions, the dual-channel micro-injection pump was used to inject different combinations of buffers through the two inlets of the mixer. At the outlet, the pH of the mixing solution at different Re was tested using a pH meter. To ensure the consistency of the results, all the tests were started one minute after the injection of the solution, and tests were repeated three times. The pH curves of the mixed buffer solutions under different Re are shown in Figure 9. From the test results, it can be seen that the pHs of the three different buffer solution combinations fluctuated above the reference value with the change of Re. When Re ≤ 0.5, the pH gradually deviated from the reference value with the increase of Re, which indicated that the mixing efficiency became worse. When Re ≥ 1, the pH gradually decreased towards the reference value with the increase of Re, which indicated that the mixing efficiency was improved. It can be seen that when 0.5 < Re < 1, the mixing efficiency turned over, which was consistent with the concentration variance curves shown in Figure 7. Additionally, when Re = 10, pH_AVG1_ = 5.01, pH_AVG2_ = 5.14, and pH_AVG3_ = 7.75 were all closest to the reference value.

**Figure 10:**
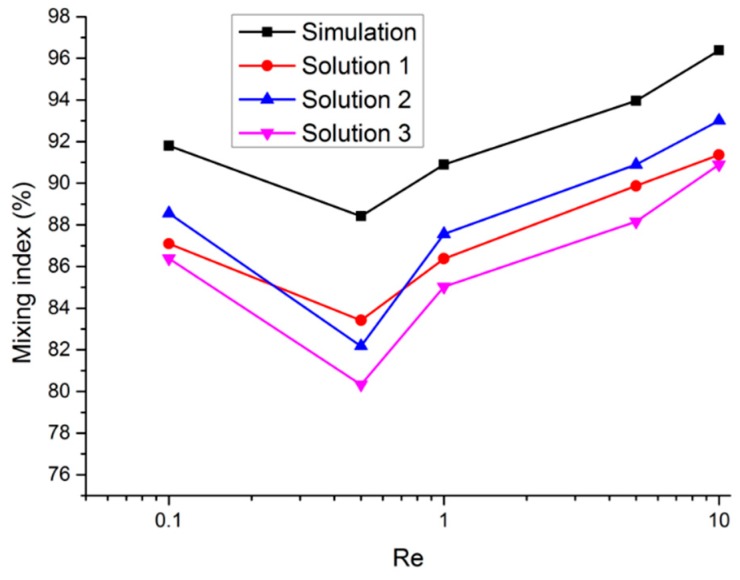
Simulated and experimentally derived mixing indexes for various Re.

The mixing index (*α*) was obtained by Equation (17), and then the relationship curves between the mixing index and Re were plotted, as shown in Figure 10. Here, *σ_max_* was the maximum concentration variance at the inlet of the mixer (unmixed). Since the Re increment was large, a log scale was used on the variation trend curves. The logarithmic scale not only compressed the size of the independent variable but also made the change trend more obvious.

(17) $$\alpha =1-\sqrt{\frac{\sigma^{2}}{\sigma_{\max}^{2}}}$$

As shown in Figure 10, when Re ≤ 0.5, the curve decreased as Re increased. When Re = 0.5, at the nadir of each curve, the simulated mixing index *α_S-min_* = 88.42%, while the experimentally derived mixing indexes *α_E-min1_* = 83.42%, *α_E-min2_* = 82.18%, *α_E-min3_* = 80.33%, respectively, with the average of the three experimentally derived values being *α_AVG-min_* = 81.98%. Here, the molecular diffusion became insufficient due to the shortened contact time as Re increased, and the convection diffusion caused by chaotic flow still did not play a leading role in the mixing. When Re ≥ 1, the mixing index curves increased as Re increased. When Re = 10, the best mixing efficiency was achieved, with the simulated mixing index being *α_S-max_* = 96.4% and the experimentally derived mixing indexes being *α_E-max1_* = 96.38%, *α_E-max2_* = 91.36%, and *α_E-max3_* = 93.01%, respectively, giving an average experimentally derived value of *α_AVG-max_* = 91.75%. Here, the mixing was mainly controlled by the convective diffusion induced by the chaotic flow. The experimentally derived mixing index curves agreed well with the numerical simulation; however, the experimentally derived values were slightly lower. Compared with the simulation results, the *α_AVG-min_* was lower by 6.44%, and the *α_AVG-max_* was lower by 4.65%. This was due to the fact that the simulation was based on ideal molecular diffusion, as well as the fact that unfavorable factors of fluid infiltration, bubbles within the fluid, and fluid polarity were not considered.

### 4.3. Performance Comparison

Finally, Table 3 lists the results of experimental tests using some micromixers that were also used for water-based fluids with Newtonian behavior. In order to directly compare the working conditions of these mixers, the flow rate or the volumetric flow that was stated in the literature was converted to Re. As can be seen from Table 3, when Re < 10, all the mixers could work. When Re < 1, more than half of the mixers worked (the mixers in references [11,12,13,14,24,26] and the mixer used in this paper), which indicated that these devices were suitable for laminar flow mixing. For all the mixers, the maximum mixing length was no more than 13 mm [12], while the minimum mixing length was 2.75 mm [11], which indicated that the structure size was suitable for integration in μTASs. Except for 3D SAR mixer (split-and-recombine) [32], the mixers shown in Table 3 consisted of more than one mixing unit, with the maximum number of units being 20 (in the mixer in reference [13]). Using a larger number of mixing units could improve the fluid chaos and thus obtain a better mixing index. However, when more units were used, the manufacturing cost and the difficulty of integration increased. Therefore, the number of mixing units was usually around 10 or fewer than 10. The mixing indexes of the various mixers are compared in Table 3. The mixing index of each mixer exceeded 70%. However, for seven mixers, the mixing index exceeded 80% (the mixers in references [11,13,14,15,24,26], and for three mixers, it exceeded 90%, namely the GSMMT (grooves staggered in the upper and lower layers at the midstream positions) [15] (*α* = 90%, when Re = 96), the 3D Tesla [13] (*α* = 94%, when Re = 1), and the 3D HT used in the present study (*α_simulation_* = 96.4%, *α_test_* = 91.75%, when Re = 10). However, compared to the 3D HT mixer, the GSMMT structure required a higher Re, while the 3D Tesla needed more mixing units to ensure mixing efficiency. The designs of the micromixers in references [8] and [9] are also based on the Horseshoe Transformation. The mixing index of the “squeeze back” HT mixer was approximately 80% (Re = 3) [24], and that of the “classic HT” was 84% (Re = 10) [26]. For the 3D HT mixer used in this paper, the simulation results revealed that the mixing index could reach 96.4% when Re = 10. However, the mixing index obtained in the experimental pH test was only 91.75% due to the non-ideal molecular diffusion and unfavorable factors (fluid infiltration, bubbles within the fluid, fluid polarity, and Joule heating). However, the mixer still achieved a good performance. It can be seen that, compared to the existing design related to the Horseshoe Transformation, the performance of the micromixer based on the 3D Horseshoe Transformation was significantly improved.

## 5. Conclusions

In this paper, a novel mixing mechanism based on the classic Horseshoe Transformation, named the three-dimensional Horseshoe Transformation (3D HT), was proposed for a passive micromixer. The Lyapunov exponent of the 3D HT, which was greater than that of the classic HT, provided strong evidence of chaotic enhancement. The structure of the mixing chamber of the 3D HT mixer, which consisted of a T-shaped inlet channel for squeezing and stretching and contained six mixing units for folding, inverse transformation, and intersection, was 12 mm in length. Numerical simulations not only proved the existence of chaotic flow in the mixer but also calculated a mixing index *α* = 96.4% (*σ* = 0.0181, Re = 10), which was a clear enhancement compared to the mixer based on the classic HT (*σ* = 0.054, Re = 10) [26]. Finally, a visualization experiment and the adjustment of pH buffer solutions were conducted to test the performance of the mixer. Microscopic images clearly showed that the mixing progress was similar to that in the simulated top-view of the concentration distribution. Although the mixing indexes of the pH buffer solutions were somewhat reduced, the 3D HT mixer (*α_AVG-min_* = 81.98% < *α* < *α_AVG-max_* = 91.75%) still performed well over a wide range of Re (0.1 < Re < 10). In future work, optimization involving more structural parameters is expected to further enhance the performance of the 3D HT mixer.

## Figures and Tables

**Table 1:** A comparison of simulation accuracy for different numbers of mesh elements.

Mesh Level	Element Size (mm)	Element Number	Curvature Factor	Relative Error
Coarser	0.103–0.334	5.3 × 10^3^	0.8	34.45%
Coarse	0.0771–0.257	12.7 × 10^3^	0.7	12.26%
Normal	0.0514–0.172	39.8 × 10^3^	0.6	7.57%
Fine	0.0257–0.136	87.2 × 10^3^	0.5	5.52%
Finer	0.0103–0.0951	254.6 × 10^3^	0.4	5.43%

**Table 2:** The composition and the pH of reference solutions.

Number	Composition	pH
1	Potassium hydrogen phthalate and borax	4.97
2	Potassium hydrogen phthalate and mixed phosphate	5.11
3	Mixed phosphate and borax	7.71

**Table 3:** A summary of chaotic micromixer designs and performances reported in the literature.

Mixer Structures (ref)	Working Condition	Mixing Length (mm)	Number of Mixing Units	Best Mixing Index
3D SAR [32]	1.5 ≤ Re ≤ 22.5	4.8	1	76% (Re = 3)
3D Serpentine [11]	0.1 ≤ Re ≤ 120	2.75	10	88% (Re = 30)
3D Twisted [12]	0.36 ≤ Re ≤ 36	12.8	8	75% (Re = 36)
3D X-shaped [14]	0.2 ≤ Re < 40	7.2	11	89% (Re = 0.2)
3D L-shaped [15]	8 ≤ Re ≤ 160	7.2	4	Above 70% (Re = 16)
GSMMT * [15]	8 ≤ Re ≤ 160	7.2	4	90% (Re = 96)
3D Tesla [13]	0.1 ≤ Re ≤ 100	11.2	20	94% (Re = 1)
“Squeeze Back” HT [24]	0.3 ≤ Re ≤ 3	3.2	8	80% (Re = 3)
Classic HT [26]	0.1 ≤ Re ≤ 50	12	4	84% (Re = 10)
3D HT [this paper]	0.1 ≤ Re ≤ 10	12	6	Simulation: 96.4% (Re = 10) Experiment: 91.75% (Re = 10)

* GSMMT: 3D grooves staggered in the upper and lower layers at the midstream positions.
